# Spanish Melon Landraces: Revealing Useful Diversity by Genomic, Morphological, and Metabolomic Analysis

**DOI:** 10.3390/ijms23137162

**Published:** 2022-06-28

**Authors:** Alejandro Flores-León, Clara Peréz Moro, Raul Martí, Joaquin Beltran, Salvador Roselló, Jaime Cebolla-Cornejo, Belen Picó

**Affiliations:** 1 COMAV, Instituto de Conservación y Mejora de la Agrodiversidad, Universitat Politècnica de València, Cno. de Vera, s.n., 46022 València, Spain; alfloleo@doctor.upv.es (A.F.-L.); clapemo@alumni.upv.es (C.P.M.); mpicosi@upv.es (B.P.); 2 Joint Research Unit UJI/UPV—Improvement of Agri-Food Quality, Universitat Politècnica de València, Cno. de Vera, s.n., 46022 València, Spain; raumarre@upv.es; 3 Instituto Universitario de Plaguicidas y Aguas (IUPA), Campus de Riu Sec, Universitat Jaume I, Avda. Sos Baynat s/n, 12071 Castellón, Spain; joaquim.beltran@qfa.uji.es; 4 Joint Research Unit UJI/UPV—Improvement of Agri-Food Quality, Department de Ciències Agràries i del Medi Natural, Universitat Jaume I, Avda. Sos Baynat s/n, 12071 Castellón, Spain; rosello@uji.es

**Keywords:** GBS, SNPs, *Cucumis melo* L., flavour, breeding, fruit quality

## Abstract

Spain is a secondary centre of the diversification of the melon (*Cucumis melo* L.), with high diversity represented in highly appreciated landraces belonging to the Flexuosus and Ibericus groups. A collection of 47 accessions of Flexuosus, Chate, Piel de Sapo, Tendral, Amarillo, Blanco, and Rochet was analysed using a genotyping-by-sequencing (GBS) approach. A total of 66,971 quality SNPs were identified. Genetic analysis differentiated Ibericus accessions and exotic materials (Ameri, Momordica, Kachri, and Agrestis), while Flexuous accessions shared ancestry between them. Within the Ibericus group, no clear genomic distinction could be identified for the different landraces evaluated, with accessions of different landraces showing high genetic similarity. The morphological characterization confirmed that the external colour and fruit shape had been used as recognition patterns for Spanish melon landraces, but variability within a landrace exists. Differences were found in the sugars and acid and volatile profiles of the materials. Flexuosus and Chate melons at the immature commercial stage accumulated malic acid and low levels of hexoses, while Ibericus melons accumulated high contents of sucrose and citric acid. Specific trends could be identified in the Ibericus landraces. Tendral accumulated low levels of sugars and citric acid and high of malic acid, maintaining higher firmness, Rochet reached higher levels of sugars, and Amarillo tended to lower malic acid contents. Interestingly, high variability was found within landraces for the acidic profile, offering possibilities to alter taste tinges. The main volatile organic compounds (VOCs) in Flexuosus and Chate were aldehydes and alcohols, with clear differences between both groups. In the Ibericus landraces, general trends for VOC accumulation could be identified, but, again, a high level of variation exists. This situation highlights the necessity to develop depuration programs to promote on-farm in situ conservation and, at the same time, offers opportunities to establish new breeding program targets and to take advantage of these sources of variation.

## 1. Introduction

The melon (*Cucumis melo* L.) is an important crop belonging to the Cucurbitaceae family, with a total global production of 28.5 million tons for the year 2020, with a considerable increase (>40%) over the last two decades [1]. Spain is the leading producer in Europe with more than 610.000 t [1], but more importantly, it represents a valuable centre of diversification. Early studies based on archaeological findings suggested Egypt or the Middle East as the domestication centres of the species [2]. However, recent molecular studies propose two independent domestication events in Africa and Asia, with most of the present melon types being derived from the Asian lineages [3]. Accordingly, India is widely accepted as the primary diversification centre of the species, and Mediterranean and Far-East diversity would have been derived by divergent diversification [4]. Among secondary centres of diversity, Spain stands out with the Ibericus group of the former Inodorus classification [5]. Nonetheless, the first melon type cultivated in the area belonged to the Flexuosus group, non-sweet melons, which were cultivated since Roman times as mentioned by roman author Columella [6,7]. Sweet melons were later introduced into the Iberian Peninsula by Muslims, who arrived in the VIII century, where they were documented as being grown by the second half of the XI century [8]. A great diversity of sweet melons was generated during centuries of cultivation, but these landraces have become progressively replaced by F1 hybrids mainly belonging to the Piel de Sapo and Amarillo market classes. Few works have analysed in depth a wide collection of Spanish landraces. In some cases, such as in Piel de Sapo commercial cultivars, melons resemble externally the corresponding landraces, but in general, a wider diversity in morpho-agronomical traits is found in Spanish landraces [9]. Nonetheless, despite the external resemblance, consumers appreciate the differences between them and value the sensory characteristics of the traditional landraces over the commercial cultivars available [10].

Few studies have analysed the diversity present in different populations of the same landrace. One example would be the simple sequence repeats (SSRs) analysis, performed by [11], of several Spanish landraces. This study revealed a higher degree of homogeneity in some types, e.g., Piel de Sapo, while others, including Rochet or Amarillo, showed higher diversity and population diversification. Other molecular studies have focused on the evaluation of a wide diversity representing worldwide landraces. Lazaro et al. [12] studied the variability of 62 Spanish melon landraces employing both SSR and morphology and identified several non-climacteric landraces, i.e., Piel de Sapo, Mochuelo, Tendral, Amarillo (Yellow)/Blanco (White), and Negro (Black), and a set of highly variable climacteric ones. Lazaro et al. [12] also pointed out that many accessions could not be classified, attesting to the great variability in Spanish melons. Other molecular studies have focused on the evaluation of a wide diversity representing worldwide landraces. With this approach, a resequencing of the transcriptome was performed of 67 melon genotypes representing the diversity grown in the World and including specific populations of specific Spanish landraces [13]. The SNP analysis revealed that higher levels of variation are still present in landraces compared to the commercial types. 

Esteras et al. [14] characterized with 768 Single Nucleotide Polymorphisms (SNPs) a collection of 68 accessions, representing global diversity and revealed that genomic regions distinguished two main groups of accessions belonging to Inodorus and Cantalupensis, the former being more variable. The group of Spanish Inodorus landraces displayed a substantial degree of polymorphism, with Blanco and Tendral being more variable than Amarillo and Piel de Sapo. Rochet accessions were separated from the rest of the accessions and Tendral was more similar to Eurasian landraces.

Genotyping-by-sequencing (GBS) technology has recently provided deeper insight into the study of melon molecular diversity and relationships, as the number of SNP studies increased dramatically. This technology has been widely employed as it is low-cost, simple, and efficient when compared to other genotyping techniques [15]. In the last decade, the evolution of GBS has enabled the number of SNPs detected and studied to multiply by 30 and has offered a complete view of almost the whole genome. Gonzalo et al. [4] and Hyun et al. [16] obtained sets of informative SNPs of 6,158 and 6,406, respectively, while Moing et al. [17] identified a global collection of 23,931 polymorphisms. A similar level of polymorphism can be detected with a local set of diversity. That would be the case of the study performed by Pavan et al. [18], which detected 25,422 SNPs in a set of Italian landraces from Apulia. All these studies used ApeKI as restriction enzyme, thus the use of an alternative enzyme would enable the analysis of different genomic regions.

The objective of the present study is to apply GBS technology to perform an in-depth study on the characteristics of a melon germplasm collection, representing the wide biodiversity of Spanish landraces, both at the intervarietal and intravarietal levels. An exhaustive characterization of morphological traits and internal quality, including the sugar and acid and volatile profiles, was also developed in order to combine data from the molecular and metabolomic point of view. The results obtained offer valuable information to establish targets for the development of next-generation cultivars with outstanding quality.

## 2. Results

### 2.1. GBS Analysis

The results for the GBS mapping indicated a correct alignment (Supplementary Table S1). On average each accession had a total of 3M reads, with an average mapping rate of 73.95%, with the lowest mapping rate obtained with the Chate accession 41CHA (71.93%) and the highest with the introgression line calc8-1 (75.07%). The Freebayes software revealed a total of 96,267 raw SNP positions, resulting in 66,971 after filtering with Vcftools for minor allele frequency (maf) of 1%, and a maximum missing count of 4. The SnpEff program revealed that 95 SNPs presented a high impact (e.g., SNPs resulting in a stop codon, loss or gain, and affects splice acceptor and donor sites), 2116 SNPs presented a moderate impact (e.g., SNPs codon changes resulting in a different amino acid), and 2858 SNPs presenting a low impact (e.g., synonymous changes) (Supplementary Table S1).

### 2.2. Population Structure Analysis

**Figure 1:**
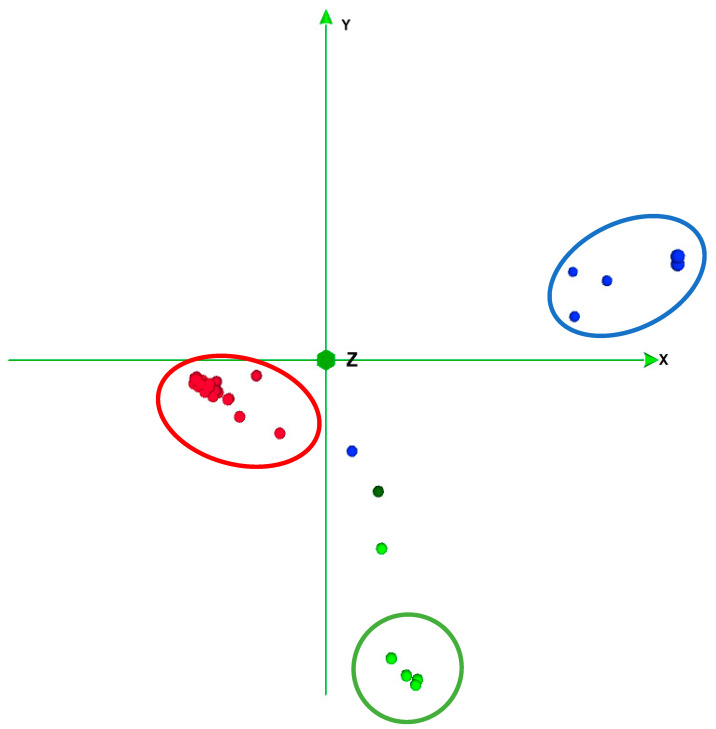
Principal component analysis (PCA) results for the Ibericus (red), exotic (blue), Flexuosus (green), and Chate (dark green) accessions studied. Subpopulations identified with ADMIXTURE (K = 3) are encircled.

The principal component analysis (PCA) biplot (Figure 1), with 29.45% of the variance explained, revealed three groups. Accessions from exotic melon groups were clearly separated. Accessions from Ibericus landraces appeared grouped with low levels of variability. Higher variability was observed in Flexuosus accessions, with accession 39AL plotting close to the Chate accession and the Ameri accession I156, with low values of the second PC.

**Figure 2:**
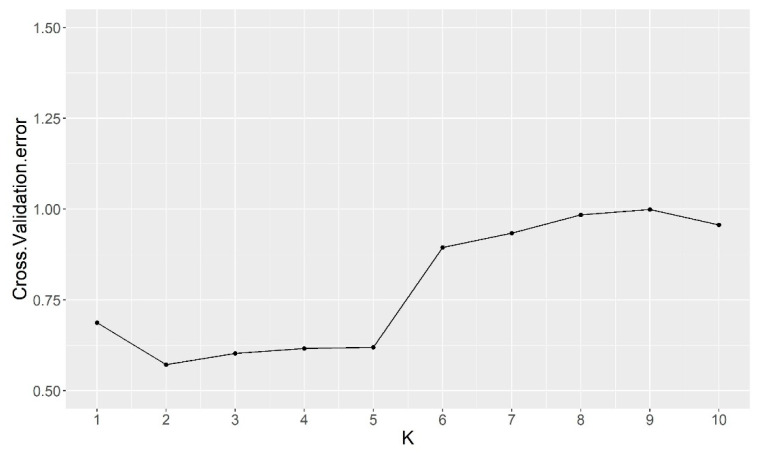
ADMIXTURE analysis results for the best grouping number based on the cross-validation error.

**Figure 3:**
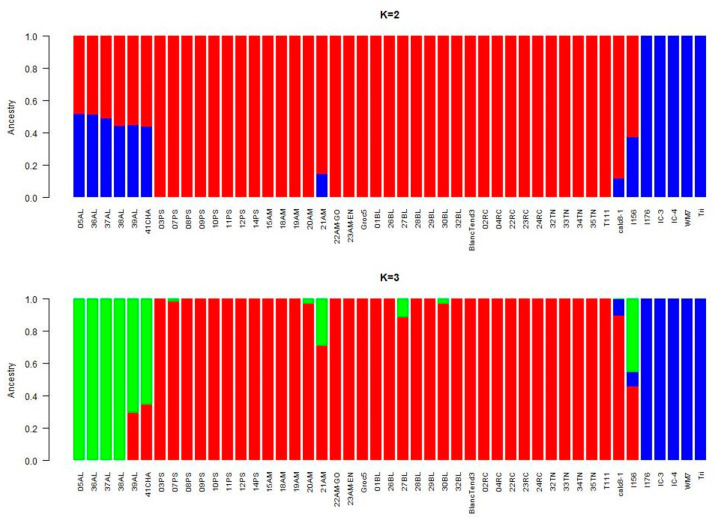
ADMIXTURE results assuming K = 2 and K = 3 populations. Each colour represents the ancestry component. Stacked bars represent each of the accessions studied.

The population structure analysis performed with ADMIXTURE software revealed that the best 2 K subpopulations values were K = 2 and K = 3 (Figure 2 and Figure 3). The bar graph showing the ancestry of each subpopulation can be seen in Figure 3. With K = 2, 2 clear subpopulations one representing the exotic types (blue) and the other with the Spanish sweet Ibericus melon accessions (red). Flexuosus and Chate accessions showed a mixed percentage (around 50%) of each subpopulation. This result agreed with the PCA results, leading to the idea that these materials stand between cultivated sweet melons and exotic wild-type melons. Accession I156 of the Ameri group also displayed a mixed ancestry pattern. Interestingly, the accession 21AM of the Amarillo landrace also displayed around 15% exotic ancestry. When a value of K = 3 was applied sweet melons and exotic materials were still recognized as different subpopulations and this time a new group was formed by the Flexuosus accessions. Exotic melon accessions still remain as part of the same group, but some changes occur. The accessions 41CHA of Chate and 39AL of Flexuosus showed a mixed ancestry between Flexuosus and sweet melons (30–35%). Specific Ibericus accessions from different landraces, 30BL and 27BL from Blanco, 07PS from Piel de Sapo, and 20AM and 21AM from Amarillo showed low levels of ancestry of the Flexuosus group. In the case of 21AM accession which previously displayed 15% exotic ancestry, this time presented a 30% ancestry from the Flexuosus group. Finally, I156 displayed again a mixed ancestry, but mainly of the sweet melon and Flexuosus group. In the PCA, the three subpopulations appeared clearly distinguished, while the Chate accession, the Algerian Flexuosus accession, and the Ameri accession plotted between the three groups, the first two closer to the Flexuosus group (Figure 1).

### 2.3. Linkage Disequilibrium Decay

**Figure 4:**
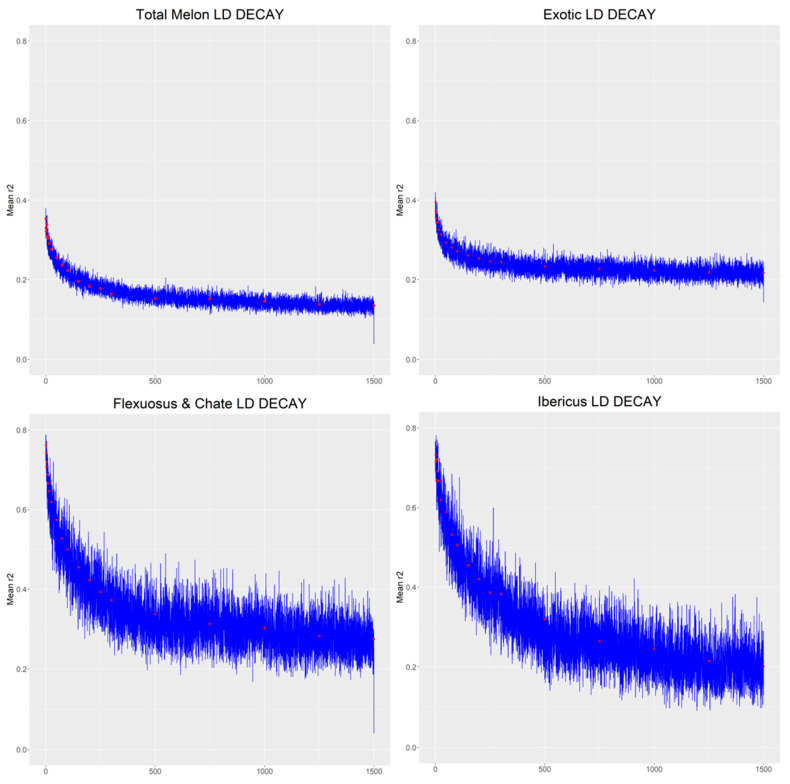
Graphs for the linkage disequilibrium in the melon populations analysed based on their distance (kb). The decay linkage disequilibrium (LD) is up to 1500 kbp. Separate graphs were created for the complete melon population, the Ibericus melons, the Flexuosus and Chate melon, and the exotic accessions.

The linkage disequilibrium (LD) decay was calculated for the complete accession collection and separately, in three different groups, Ibericus, exotic and Flexuosus, and Chate melons (Figure 4). The LD was variable among the different groups. Overall, the results showed a much faster decay in exotic melons. When the whole collection was analysed, the LD decay distance (r^2^ = 0.2) was approximately 100 kbp. Within groups, the decay distance of exotic melons LD (r^2^ = 0.25) remained around 100 kbp, but it was higher for Ibericus melon (300 kbp; r^2^ = 0.30) and Flexuosus and Chate melons (300 kbp; r^2^ = 0.35).

### 2.4. Phylogeny

**Figure 5:**
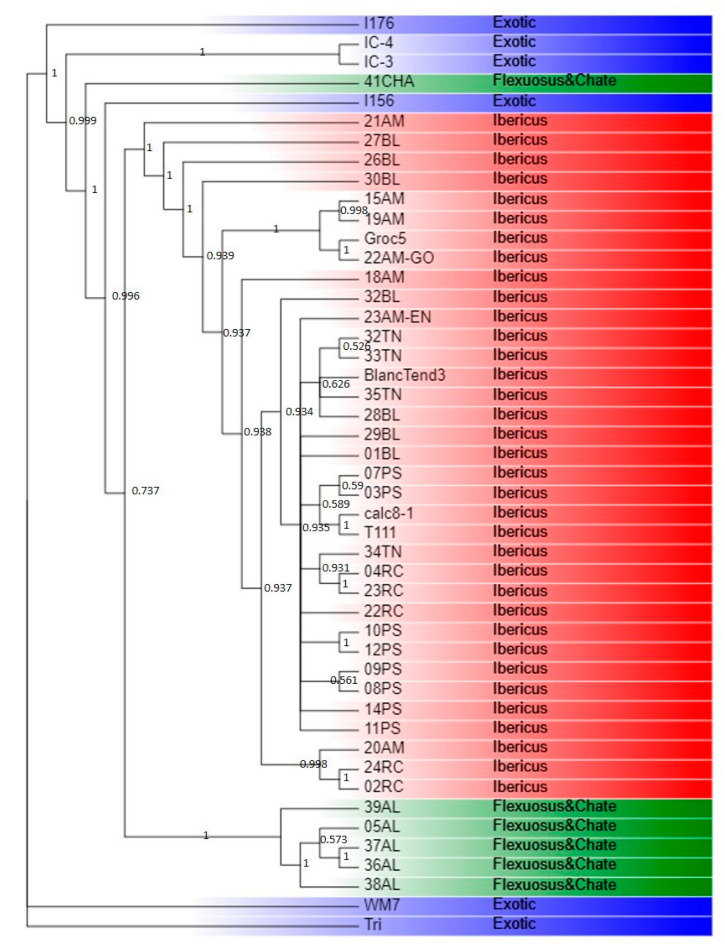
Consensus maximum likelihood phylogenetic tree (50% cut-off), representing the phylogenetic relationships of the Ibericus melon (Red), exotic (Blue), Flexuosus, and Chate (Green). Trigonus serves as the outgroup of the tree. Bootstrap values are based on 1000 iterations.

The consensus phylogenetic tree (Figure 5) separated Agrestis and Kachri melons from the rest of the accessions. Momordica accessions IC-3 and IC-4 were closely grouped and at a certain distance from the remaining Momordica accession I176. The exotic accession I156 from the Ameri group and the Spanish landraces were grouped separately and formed four clades, one with the Chate accession, one with I156, another one with the Flexuosus accessions, and the remaining one with the Ibericus accessions. Within the Flexuosus clade, Spanish landraces appeared grouped together and at a certain distance from the Algerian accession 39AL. Within the Ibericus clade, accessions from different landraces appeared intermixed. Only in some cases, some accessions of certain landraces configured specific groups. For example, 15AM, 19AM, Groc5, and 22AM-GO from the Amarillo landrace were grouped together and at a certain distance of 21AM and 18AM. In the rest of the cases, Tendral, Blanco, Rochet, and Piel de Sapo accessions appeared intermixed. Only certain couples of accessions of the same landrace appeared grouped with high bootstrap values and low genetic distance.

### 2.5. Fruit Characterization

**Figure 6:**
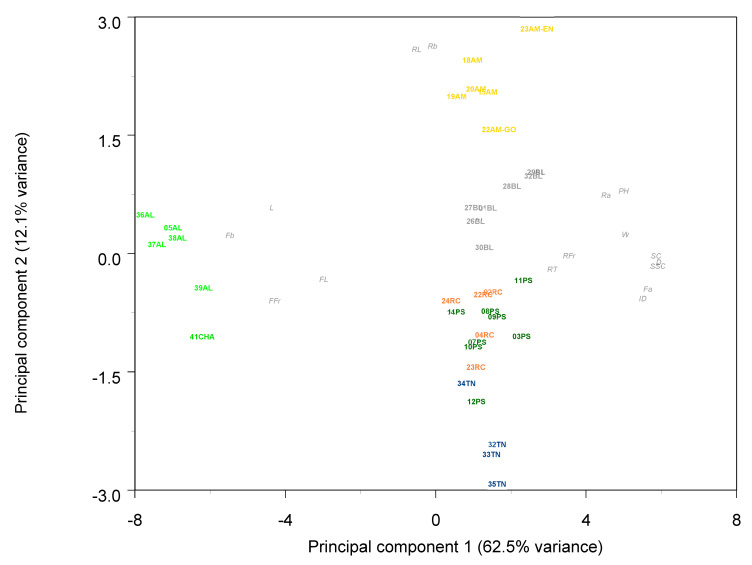
Biplot of principal component analysis of morphological traits. AL: Flexuosus; CHA: Chate; PS: Piel de Sapo; TN Tendral; AM: Amarillo; BL: Blanco; RC: Rochet. Variables indicated in italics. F: Fruit; R: Rind; I: Internal; W: Weight; L: Length; D: Diameter; Fr: Firmness; SC: Seed cavity; SSC: Soluble solids content; L, a, b: Hunter colour coordinates.

**Figure 7:**
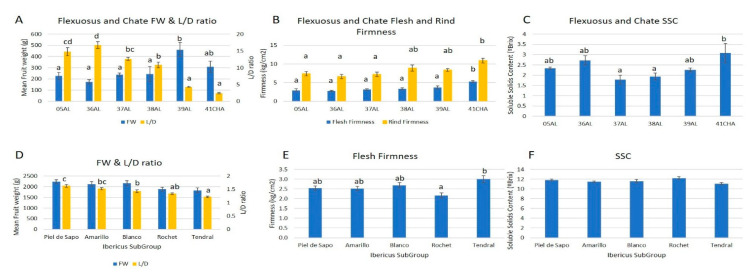
Fruit Weight, L/D ratio for the Flexuosus and Chate fruits (**A**) and the Ibericus Subgroups (**D**); flesh and rind firmness for the Flexuosus and Chate fruits (**B**) and Ibericus landraces (**E**); and soluble solids content for the Flexuosus and Chate fruits (**C**) and Ibericus landraces (**F**). Different letters indicate significant differences (Tukey’s test, *p* ≤ 0.05). Those bars without letters had no significant differences in the ANOVA test (*p* = 0.05) and no differences were found between landraces.

Fruits are characterized at their commercial state. Consequently, Flexuosus and Chate fruits were collected whilst immature, while Ibericus fruits were harvested when ripened. High variability was present between and within groups (Figure 6 and Figure 7, Supplementary Table S2). Nonetheless, the principal component analysis (Figure 6) revealed, as expected, a clear differentiation between Flexuosus and Chate accessions and those belonging to the Ibericus group. The former had long and narrow green fruits with no seed cavity and a minimum rind and these variables differentiated both groups. As these fruits are eaten immature, flesh firmness was also higher than in the Ibericus group. The overall fruit weight of the Flexuosus–Chate group ranged between 170 and 450 g. In general, Chate could be differentiated as having an intermediate weight (309 g) and shorter fruits, with the smallest L/D ratio. Among Flexuosus accessions, the only ribless accession, 39AL from Algeria, presented the highest weight but these fruits were shorter than the rest of the Flexuosus accessions. The fruit pH of Flexuosus and Chate melons was similar (4–4.7), but Chate accession showed higher SSC values compared to Flexuosus accessions (3.1°Brix vs. 1.8–2.7°Brix). 

Landraces of the Ibericus group were mainly differentiated by rind colour (Figure 6, Figure 7, Supplementary Table S2), being white for Blanco melons, yellow for Amarillo, and greenish for Piel de Sapo, Tendral, and Rochet. Regarding size, fruits from the Ibericus group showed similar sizes with mean fruit weight ranging between 1800 and 2200 g. Within each landrace, weight was similar, although Blanco accessions showed higher variability. In this landrace, accessions 26BL and 01BL (1250–1650 g) presented significantly lower fruit weight than 30BL (2815.5 g). Tendral and, to a lesser extent, Rochet melons tended to show a lower length to diameter ratio with a more rounded shape than the rest of the landraces, which were more elongated, especially Piel de Sapo. General trends were identified for each landrace, though in some cases (e.g., 23AM-EN in Amarillo or 34TN in Tendral) certain accessions tended to show differences from the rest of the group. In the case of Piel de Sapo and Rochet the differences were less marked, and the spectra of variation overlapped. 

Nonetheless, Rochet melons tended to be less firm (Flesh Firmness = 2.2 kg/cm^2^), Piel de Sapo, Amarillo, and Blanco showed intermediate values and Tendral fruits were firmer (3 kg/cm^2^). Within each landrace, variability was found in Piel de Sapo and Blanco for flesh firmness (accessions 12PS and 30BL being firmer). On the other hand, no significant differences were detected for SSC between landraces with values ranging between 11 and 12.25°Brix. Nonetheless, within Piel de Sapo and Blanco landraces significant differences were detected between accessions with 08PS, 03PS, and 27BL), reaching 1.5–2°Brix values lower than 11PS and 28BL. 

The rind colour varied between the greenish landraces. Piel de Sapo accessions presented green colour with darker green spots, Rochet displayed a light green external colour, and Tendral presented a darker green rind colour. In Amarillo melons, external colour was rather uniform, but differences were found within landrace flesh Hunter colour coordinates (red–green gradient). In the case of the Blanco landrace, accessions 28BL and 29BL displayed green coloured stripes in sections of their rind of varying lengths. In the last two landraces, significant differences were found between accessions for seed cavity diameter.

### 2.6. Sugars and Acids Content

**Figure 8:**
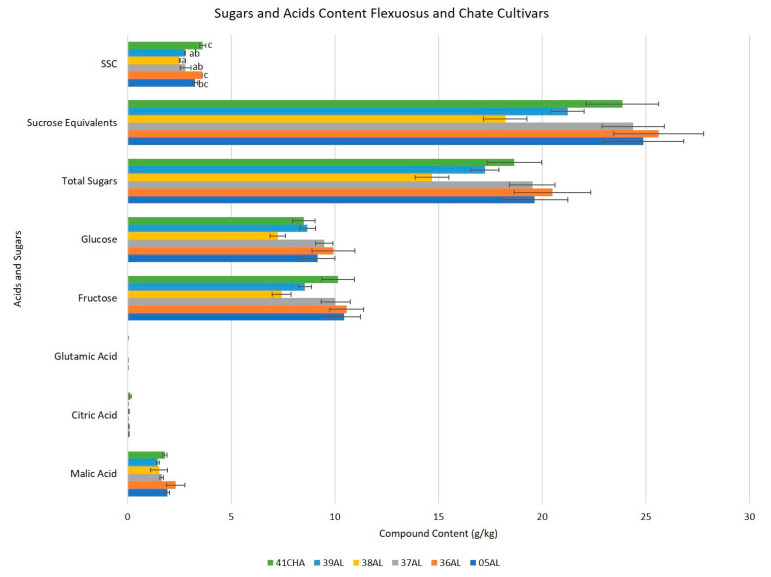
Mean acids and sugar content for the Flexuosus and Chate accessions. Different letters indicate significant differences (Tukey’s test, *p* ≤ 0.05). Those bars without letters had no significant differences in the ANOVA test (*p* = 0.05) and no differences were found between landraces.

The main difference between the Flexuosus–Chate and Ibericus groups was that the former accumulated lower levels of sugars and did not accumulate significant contents of sucrose (Figure 8, Supplementary Table S3). Additionally, in the former group, the predominant acid was malic acid, while in the Ibericus group it was citric acid, and glutamic acid levels in the former were close to the limit of quantification. Within the Flexuosus–Chate group, a trend towards higher SSC was confirmed for Chate, though no significant differences were found between this population and 36AL from Flexuosus. Nonetheless, no significant differences were found for hexoses and acid accumulation between accessions of the group.

**Figure 9:**
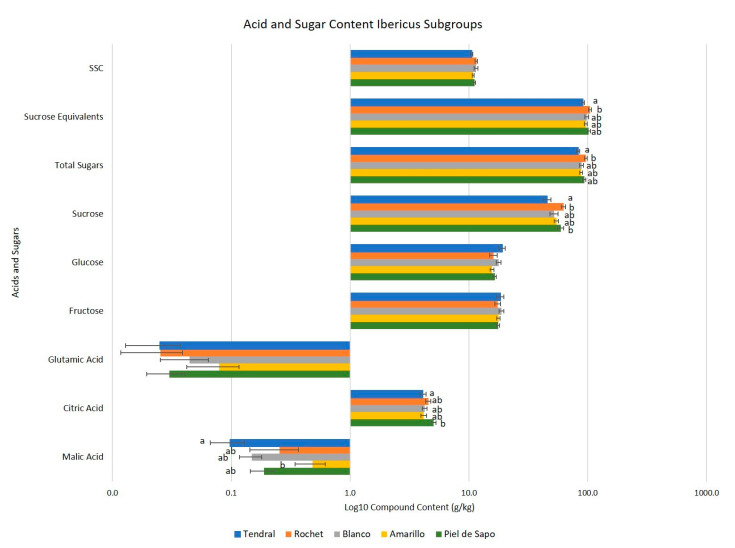
Mean acids and sugar content for the Ibericus landraces. Different letters indicate significant differences (Tukey’s test, *p* ≤ 0.05). Those bars without letters had no significant differences in the ANOVA test (*p* = 0.05) and no differences were found between landraces.

In the case of the Ibericus group (Figure 9, Supplementary Table S3), differences were found between landraces for both the acid and sugar contents. Tendral presented the highest malic acid content and the lowest citric acid levels. On the other hand, Amarillo tended to offer low malic acid contents. In the case of sugar content, as expected, the main sugar was sucrose, with lower contents of fructose and glucose. Rochet and Piel de Sapo had the highest sucrose levels, but the higher accumulation of fructose of the former resulted in higher values of total sugars and sucrose equivalents. Within landraces, special considerable differences were found in acid contents (Supplementary Table S3). Overall, no significant differences were observed in the content of both fructose and glucose between the different accessions of each landrace but in the Rochet landrace, accessions 02RC and 22RC offered significantly lower.

### 2.7. Volatile Organic Compouds (VOCs) Content

**Figure 10:**
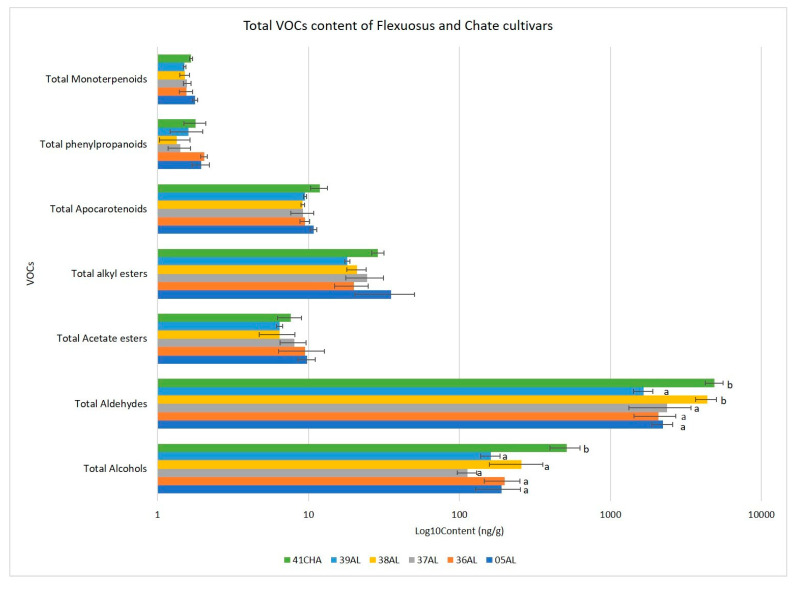
Total VOCs content for the Flexuosus and Chate accessions. Different letters indicate significant differences (LSD test, *p* ≤ 0.05). Those bars without letters had no significant differences in the ANOVA test (*p* = 0.05) and no differences were found between landraces.

**Figure 11:**
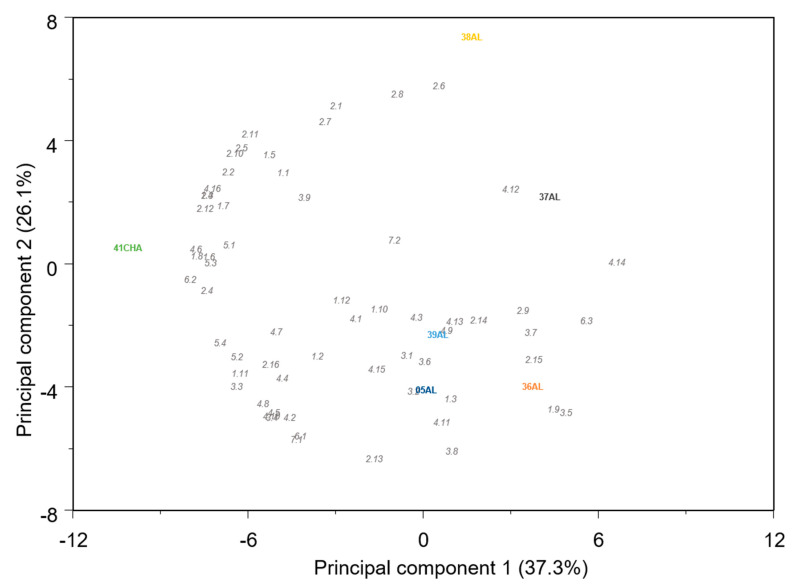
Principal component analysis biplot for the VOCs profile of the Flexuosus and Chate melon accessions. 1_1(1-pentanol); 1_2(1-hexanol); 1_3((Z)-3-hexen-1-ol); 1_4(1-octanol); 1_5(1-nonanol); 1_6((Z)-3-nonen-1-ol); 1_7((Z)-6-nonen-1-ol); 1_8((E,Z)-2,6-nonadien-1-ol); 1_9(1-decanol); 1_10(benzyl alcohol); 1_11(2-phenylethanol); 1_12(phenol); 2_1(Hexanal); 2_2(Heptanal); 2_3((E)-2-heptenal); 2_4((E,E)-2,4-heptadienal); 2_5((E)-2-octenal); 2_6(octanal); 2_7(nonanal); 2_8((Z)-6-nonenal); 2_9(benzaldehyde); 2_10((E)-2-nonenal); 2_11((E,Z)-2,6- nonadienal); 2_12((E,E)-2,4-nonadienal); 2_13(Decanal); 2_14((E,E)-2,4-decadienal); 2_15(phenylacetaldehyde); 2_16(2-hydroxybenzaldehyde); 3_1(amyl acetate); 3_2(butyl acetate); 3_3(benzyl acetate); 3_4(hexyl acetate); 3_5((Z)-3-hexen-1-ol-acetate); 3_6(heptyl acetate); 3_7(octyl acetate); 3_8(2-methylpropyl acetate); 3_9(phenethyl acetate); 4_1(methyl butyrate); 4_2(methyl 2-methylbutyrate); 4_3(ethyl butyrate); 4_4(ethyl 2-methylbutyrate); 4_5(propyl butyrate); 4_6(butyl isobutyrate); 4_7(isobutyl butyrate); 4_8(ethyl (E)-2-butenoate); 4_9(butyl butyrate); 4_10(isoamyl butyrate); 4_11(ethyl pentanoate); 4_12(methyl hexanoate); 4_13(ethyl hexanoate); 4_14(ethyl heptanoate); 4_15(ethyl 3-(methylthio)propanoate); 4_16((E,E)-2,4-hexadienoic acid, ethyl ester); 5_1(6-methyl-5-hepten-2-one); 5_2(beta-cyclocitral); 5_3(geranylacetone); 5_4(beta-ionone); 6_1(guaiacol); 6_2(eugenol); 6_3(isoeugenol); 7_1(linalool), 7_2(Eucalyptol).

**Figure 12:**
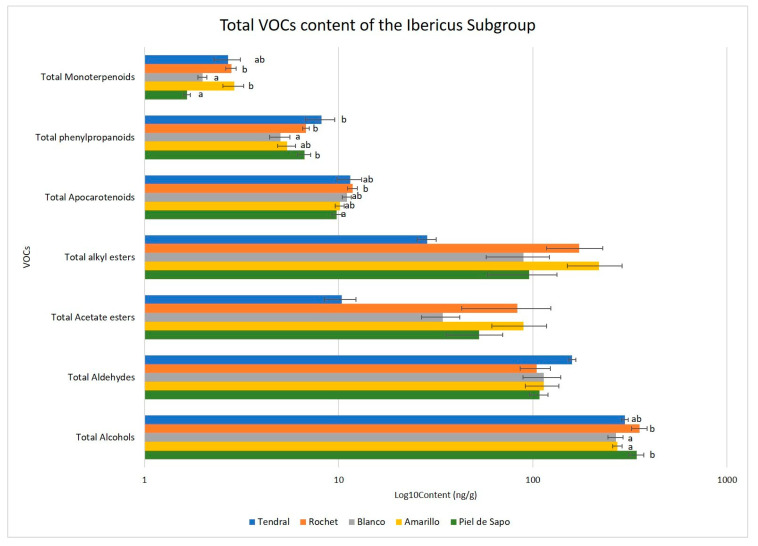
Total VOCs content for the Ibericus landraces. Different letters indicate significant differences (LSD test, *p* ≤ 0.05). Those bars without letters had no significant differences in the ANOVA test (*p* = 0.05) and no differences were found between landraces.

Important differences were found between the Flexuosus–Chate and the Ibericus group in the VOC profile. In brief, accessions from the former had increased levels of aldehydes and lower levels of esters (Figure 10 and Figure 11, Supplementary Table S5). In fact, aldehydes represented the main compounds of the Flexuosus and Chate melons aroma profile, followed by alcohols, and alkyl esters. Smaller contents of apocarotenoids and acetate esters were also detected. A small content of phenylpropanoids and monoterpenoids was also found. Among aldehydes, (E,Z)-2,6- nonadienal, (E)-2-nonenal, nonanal, and (Z)-6-nonenal showed the highest levels, while 1-nonanol, 2-phenylethanol, and (Z)-3-nonen-1-ol were the most abundant alcohols. Among this group of accessions, 41CHA and 38AL outstood for higher accumulation of aldehydes, reaching 4900.82 ng g^−1^ and 4363.67 ng g^−1^, respectively (Figure 12); contents that doubled those of the rest of the accessions of the group (1660–2300 ng g^−1^). The Chate accession 41CHA also doubled the accumulation of alcohols of Flexuosus accessions (512.95 ng g^−1^ vs. 161–256 ng g^−1^).

The PCS of VOCs contents confirmed the different volatile profiles of Chate and Flexuosus accessions, mainly explained by the differential accumulation of aldehydes and alcohols (Figure 11). Within the Flexuosus group variability was observed with two subgroups of accessions. One of them is formed by 05AL, 36AL, and 39AL, characterized by a higher content of alkyl esters, such as ethyl hexanoate or ethyl (E)-2-butenoate, acetate esters, (Z)-3-hexen-1-ol-acetate, or 2-methylpropyl acetate, and the other formed by 37AL and 38AL, with lower ester accumulation. Between these two last accessions, important differences were found though, as 38AL reached high accumulation levels of aldehydes.

**Figure 13:**
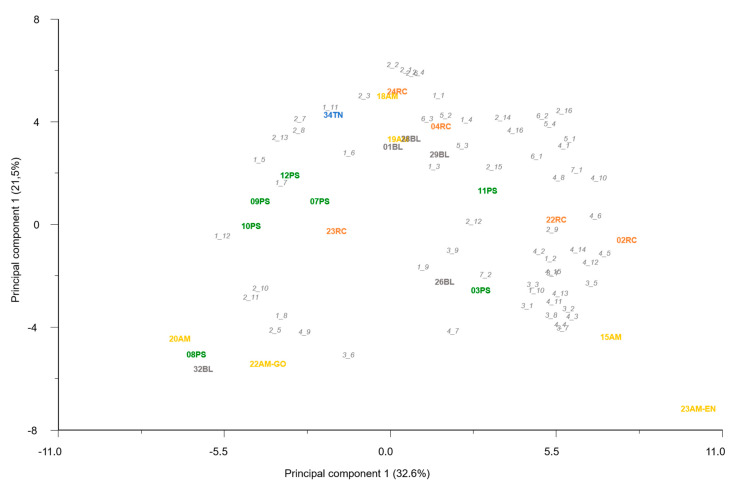
Principal component analysis for VOCs profile of Ibericus melon accessions. 1_1(1-pentanol); 1_2(1-hexanol); 1_3((Z)-3-hexen-1-ol); 1_4(1-octanol); 1_5(1-nonanol); 1_6((Z)-3-nonen-1-ol); 1_7((Z)-6-nonen-1-ol); 1_8((E,Z)-2,6-nonadien-1-ol); 1_9(1-decanol); 1_10(benzyl alcohol); 1_11(2-phenylethanol); 1_12(phenol); 2_1(Hexanal); 2_2(Heptanal); 2_3((E)-2-heptenal); 2_4((E,E)-2,4-heptadienal); 2_5((E)-2-octenal); 2_6(octanal); 2_7(nonanal); 2_8((Z)-6-nonenal); 2_9(benzaldehyde); 2_10((E)-2-nonenal); 2_11((E,Z)-2,6- nonadienal); 2_12((E,E)-2,4-nonadienal); 2_13(Decanal); 2_14((E,E)-2,4-decadienal); 2_15(phenylacetaldehyde); 2_16(2-hydroxybenzaldehyde); 3_1(amyl acetate); 3_2(butyl acetate); 3_3(benzyl acetate); 3_4(hexyl acetate); 3_5((Z)-3-hexen-1-ol-acetate); 3_6(heptyl acetate); 3_7(octyl acetate); 3_8(2-methylpropyl acetate); 3_9(phenethyl acetate); 4_1(methyl butyrate); 4_2(methyl 2-methylbutyrate); 4_3(ethyl butyrate); 4_4(ethyl 2-methylbutyrate); 4_5(propyl butyrate); 4_6(butyl isobutyrate); 4_7(isobutyl butyrate); 4_8(ethyl (E)-2-butenoate); 4_9(butyl butyrate); 4_10(isoamyl butyrate); 4_11(ethyl pentanoate); 4_12(methyl hexanoate); 4_13(ethyl hexanoate); 4_14(ethyl heptanoate); 4_15(ethyl 3-(methylthio)propanoate); 4_16((E,E)-2,4-hexadienoic acid, ethyl ester) 5_1(6-methyl-5-hepten-2-one); 5_2(beta-cyclocitral); 5_3(geranylacetone); 5_4(beta-ionone); 6_1(guaiacol); 6_2(eugenol); 6_3(isoeugenol); 7_1(linalool), 7_2(Eucalyptol).

In the landraces of the Ibericus group, the most important VOCs accumulated were alcohols, esters, and aldehydes (Figure 12 and Figure 13, Supplementary Table S4). Accessions of Piel de Sapo and Rochet landraces had significantly higher total alcohol contents than Amarillo and Blanco, but no significant differences were observed for the aldehyde or ester contents. Piel de Sapo presented the lowest total apocarotenoids and monoterpenoids, but high contents of phenylpropanoids. Tendral and Rochet presented high contents of phenylpropanoids and monoterpenoids, and Blanco presented low levels of both compounds. 

The PCA of VOCs contents in the Ibericus revealed that, despite existing general trends, the spectrum of variation of sweet melon landraces overlapped (Figure 13). One of the more variable landraces was Amarillo, which showed two types of behaviour. Accessions 20AM and 22AM-GO had higher contents of certain aldehydes and alcohols, such as (E)-2-octenal, (E,Z)-2,6- nonadienal, and (E,Z)-2,6-nonadien-1-ol, while 15AM and 23AM-EN stood out regarding ester accumulation. Specifically, 23AM-EN presented high contents of alkyl esters and, particularly, ethyl 2-methylbutyrate and ethyl 3-(methylthio) propanoate. 

In general, Rochet accessions tended to accumulate higher contents of esters and Piel de Sapo higher levels of alcohols and aldehydes, but within each landrace variability was found again. Accessions 07PS, 09PS, 10PS, and 12PS of Piel de Sapo formed a rather uniform group, with intermediate VOCs levels, while 11PS and 03PS tended to accumulate higher levels of VOCs and 08PS lower levels. In the Rochet landrace, 22RC and 02RC tended to accumulate higher levels of VOCs and, specifically, of esters, while 24RC, 04RC, and 23RC showed lower accumulation, especially the last one. The only Tendral accession analysed for VOC contents, 34TN, showed an intermediate profile between Rochet and Piel de Sapo.

**Figure 14:**
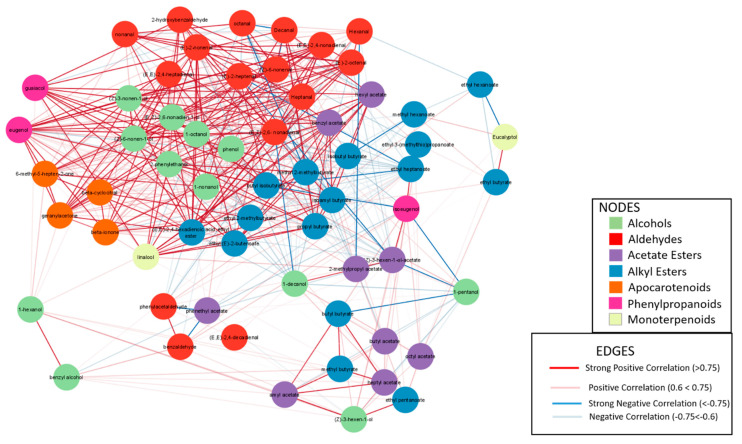
Correlation network analysis of the VOCs profile of the Flexuosus and Chate group. Each node represents one VOC. Positive and negative correlations are indicated (red for positive, blue for negative) and line thickness increases for higher correlation values.

In order to analyse the metabolomic profile of VOC accumulation, two correlation networks were obtained, considering the different volatile profiles of the Flexuosus–Chate group and Ibericus. In the Flexuosus–Chate group, strong correlations were found, in general, within alcohols, aldehydes, and alkyl esters and between these groups of volatiles (Figure 14). Within acetate esters, the correlations within group were not solid, though some of them correlated with specific alcohols and aldehydes. Apocarotenoids correlated among themselves and with certain alcohols and the phenylpropanoid eugenol. Interestingly, phenylpropanoids eugenol and guaiacol did not correlate with each other, but guaiacol correlated with several alcohols, esters and the phenylpropanoid linalool. Monoterpenoids eucalyptol and linalool did not correlated. The former correlated positively with ethyl butyrate and negatively with ethyl hexanoate, while the latter correlated with guaiacol and several esters.

**Figure 15:**
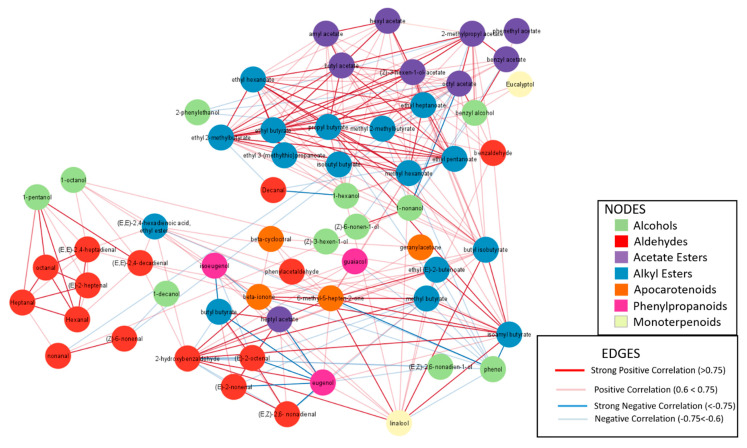
Correlation network analysis of the VOCs profile of the Ibericus group. Each node represents one VOC. Positive and negative correlations are indicated (red for positive, blue for negative) and line thickness increases for higher correlation values.

The correlation network of the Ibericus group had a different pattern (Figure 15). Two main subgroups of aldehydes could be identified, while decanal and benzaldehyde appeared dispersed among other VOCs. Alcohols also appeared scattered in relationships with aldehydes and esters. Alkyl and acetate esters, on the other hand, appeared more correlated within and between each other. The relationships between apocarotenoids were not as clear as in the Flexuosus–Chate group, but some relationships were maintained, for example with 2-methybutyrate and (E,E)-2,4-hexadienoic acid ethyl ester.

## 3. Discussion

The use of GBS technology to study and characterize melon germplasm has been widely employed with one of the most recently performed assays, employing 2083 world accessions [19]. These studies all used the ApeKI restriction enzyme, which has permitted the comparison between different datasets, as well as usage of versions 3.5.1 or 3.6.1 of Melon DHL92. The present study was performed employing a different enzyme MsII, which allowed the study of different areas of melon genome, as well as the usage of the most recent Genome v4. Despite this change, the genomic analysis offered further details but remains consistent with previous studies. 

Our present study with 47 melon accession revealed a total of 96,267 raw SNP positions, after filtering resulted in 66,971 high quality SNPs. Previous studies [20,21] have achieved different results, with a higher quantity of both raw and quality filtered SNPs. The criteria of SNP filtering do vary in different previous studies [16,19,22], with the use of different minor allele frequency (maf) of either 0.01 or 0.05, as well as different maximum missing data. With more restrictive criteria, the resulting filtered SNPs will be less in quantity. Our criteria for maf and maximum missing data would serve as to conserve SNPs, which are rare in the accessions employed, while the maximum missing enables us to have more genomic data for each position. The 66,971 SNPs filtered, as well as the use of accessions of a more exotic nature, allowed for a better study of melon landraces. 

Our study showed a clear grouping of the exotic, Ibericus, and Flexuosus melons, although the I156 Ameri melon, 41CHA Chate, and 39AL melon displayed more distance from their respective groups. Our population structure analysis corroborated our PCA results, showing K = 2 and K = 3 to be the best and second best, similar to those obtained by other authors [16,21,23,24]. Leida et al. [23] found that Ibericus melons formed their own subpopulation (K = 7, second best), while Flexuosus and Chate had mixed subpopulations without forming their own group. Abu Zaitoun et al. [24] studied the 88 Palestinian snake melon local landraces and found two different subpopulations differentiated by their geographical origin. Our results show that the Spanish Flexuosus formed a tight-knit group with the Algerian 39AL being more distant from them.

The results obtained for the phylogenetic relationships of the melon accessions clearly revealed a distancing from the more exotic accessions, including a certain proximity between the Flexuosus and the Ibericus melons, similarly to results obtained by the population structure analysis. Our results also showed that the Ameri accession I156 was phylogenetically close to the cultivated melons, which has been previously observed in other works, e.g., by Nimmakayala et al. [25] and Moing et al. [17]. In their study, Sabato et al. [26] found, using 179 melon accessions and archaeological melon seeds, that the population containing the archaeological seeds also included Ameri melons and Italian Chate, as well as Flexuosus melons. This also coincides with our results as both the Ameri and Chate melons had high genomic similarity as compared to other groups. In the Ibericus clade, some of the melons appear to be more distant than the others. These seem to be those that contain a small percentage of ancestry belonging to the Flexuosus subpopulation, again validating the results obtained in the population structure analysis. Overall, our results show no clear grouping, according to the classification performed by Pitrat [5], with some cultivars of different subgroups grouping together. The subgroups proposed by Pitrat [5] only consider the exocarp of the fruits to differentiate between them. This simplification is quite useful in a commercial sense, but not from a genetic point of view. Esteras et al. [14], in their study of 93 diverse melon accessions, examined the variability within the Spanish Inodorus landraces (which today comprise the Ibericus Group) and found that some landraces did form clear groups, such as the Piel de Sapo, but in some cases, clusters could be found which contained melons of a different type, again finding that geographical origin can play an important role, as can their morphological type. Lazaro et al. [12] were able to obtain seven groups based on their morphology, but the SSR analysis revealed two major groups, the first and largest containing Piel de Sapo, Tendral, and Winter, with some Yellow/White (with Tendral characteristics), while the second included Black, Mochuelo, and Yellow/White melons. In general, the present study, performed with a considerably higher number of SNPs, agrees with previously published information. The Ibericus accessions appeared mixed and, although some accessions of Piel de Sapo, Amarillo or Tendral tended to form groups, the truth is that accessions from different landraces appeared in the same subclusters, probably denoting a high level of shared genomes, with subtle genomic differences defining the landrace typical attributes. 

The use of snpEff permitted the discovery of several interesting SNPs with a high impact on specific genes. For example, accessions 41CHA and 39AL, presented a SNP causing a stop gain in MELO3C018465, a Glycosyltransferase, which play an important role in maintaining cell homeostasis and regulates plant growth and development [27]. MELO3C024563 (Putative UDP-N-acetylglucosamine--peptide N-acetylglucosaminyltransferase SPINDLY), MELO3C024565 (mRNA-decapping enzyme-like protein), and MELO3C015904 (SWR1-complex protein 4) have been detected to have modifications in our study, with the last one having a SNP which changes an amino acid in the sequence, mainly in the Ibericus and some of the Flexuosus melons. These genes have been previously reported by Kishor et al. [21] to have an effect on sex expression in Oriental melon (*Cucumis melo* L.var. *makuwa*). Another interesting SNP detected in all the essayed accessions was located in gene MELO3C020760, a SAUR20-like auxin-responsive protein. These genes (SAUR) are part of an important family related to auxin signal transduction, usually employed as marker genes [28]. Recent analysis have identified cotton, an SNP locus (Gh_D08G1308) associated to plant salt tolerance which resulted to belong to SAUR-like auxin-responsive protein family [29]. Tzuri et al. [30] found that SNPs *CmOr* (MELO3C005449) related to fruit quality and responsible for fruit flesh colour was not detected in our sample. Other SNP important genes, such as MELO3C010779 *(CmACS-11*), an androecy gene which controls female flower development (as can be seen in Boualem et al. [31]), also did not appear in our sample. Natarajan et al. [32] identified several Some SNPs associated with defence genes against powdery mildew that have been observed in our samples, such as MELO3C002352 (Argininosuccinate synthase). Moderate change A/C was detected in accessions 41CHA and 39AL on MELO3C022146 (TMV resistance protein N-like). A moderate effect T/C was detected on most exotic materials but also on some Ibericus melons, i.e., 32BL, 03PS, and the Spanish Flexuosus melons. Another SNP change was detected in MELO3C022339 (Glutaredoxin protein) with a moderate change in Trigonus (G/T) and a low change (C/T) in some Flexuosus, Ibericus 21AM, Trigonus, and WM7. This last change has already been linked to resistance DEGs (differential expressed genes) associated with the response to *Tomato Leaf Curl New Delhi Virus* (ToLCNDV), with the SNP being present in resistant WM7 but not in the susceptible Piel de Sapo variant Piñonet [33].

Previous studies characterizing Spanish landraces revealed that most part of the variation was focused on the external colour, shape (globular to elongate), and rind patterns [9]. In general, all of them had large sizes. As reported by Lazaro et al. [12], it seems that farmers selected large sizes that usually require longer growing cycles. Indeed, external appearance has been used in this and other crops, such as the tomato, as a key factor for the recognition of landraces by farmers and consumers, resulting in a clear differentiation in basic morphological traits [34]. In this sense, our results revealed that although some level of variation exists within landrace, they can easily be distinguished in most cases. This is evident for the Flexuosus and Chate group compared to the Ibericus landraces, as in the former, fruits are long and elongated and lack seed cavities. However, even in this group, the Chate accession evaluated presented differences with most of the Flexuosus accessions, with shorter and wider fruits. Within the Flexuosus group, however, some degree of variability was found. Soltani et al. [35] observed a high degree of variability in ribless accessions from Iran but not in the ribbed ones. In our case, only one ribless accession 39AL was evaluated and, in fact, presented a distinct phenotype compared to the ribbed materials in other traits. Nonetheless, the variability found seems to be more restricted than that described by Ali-Shtayeh et al. [36] in Palestine with different landraces. In Spain, snake melons were already described by Columella [37], but, nowadays, after a strong genetic erosion process, its cultivation is highly limited, which would explain the lower variability compared to that found in the Middle East, where they are still widely cultivated, well-known, and appreciated.

In the case of the Ibericus melon, landraces are mainly distinguished by external colour, even within greenish landraces. However, despite these general trends, it is possible to recognize specific accessions with special differentiation, as it would be the case of 23AM-EN (with a more intense yellow rind colour, longer, and more seed cavity) in Amarillo or 34TN (with a lighter green rind colour) in Tendral. Apart from external appearance, long-term conservation also can be used as a key differentiating factor. In the present study, the winter melon Tendral, with long-term conservation clearly differing from other landraces by its high flesh firmness, confirming a slow ripening pattern, which de Graça Barreiro et al. [38] linked to limiting steps in the synthesis of ethylene. Artés et al. [39] also found the highest firmness values in Tendral melons compared to other Spanish landraces, and, as concurred by de Graça Barreiro et al. [38], they described lower SSC values in this landrace. In our case, SSC values were not significantly different, though a trend towards lower values seemed evident. Apart from the evident difference in the shape of Flexuosus–Chate and Ibericus melons, the other main difference is related to their sugar and acid profile. Flexuosus are not sweet melons, and accordingly, SCC obtained in these accessions were much lower than the Ibericus accessions. In general, the values obtained were lower than those described in the similar Italian landraces Carosello and Barattiare that reach up to 3.6–4°Brix [40]. Nonetheless, these results could be a consequence of environment and genotype x environment interactions, as similar Spanish accessions grown in different environments also offered SSC values ranging from 3.3 to 4.0°Brix [7]. Burger et al. [41] described in the Flexuosus melon Faqqous the genotypic combination *Suc*/*Suc* that prevents sucrose accumulation in the fruit. Accordingly, in our analysis sucrose levels in the Flexuosus–Chate melons remained under quantification limits. 

The acidic profile also differed in this group. Both citric and malic acid were detected, but the predominant acid was malic in the Flexuosus-Chate group, while in Ibericus melons citric acid was predominant. Burger et al. [41] suggested that the *so*/*so* mutation responsible for a high acidic profile was fixed in melons before the fixation of the *suc*/*suc* mutation that led to sweet melons. Cohen et al. [42] also described that the *CmPH* allele is present in non-sweet melons and leads to substantial increases in the acidic profile at the mature stage. In our case, the levels of acids at the immature commercial stage are lower than those of Ibericus landraces at the mature stage, but the predominant acid changes. This change in the profile would be justified by the trends of acid accumulation in melon, as in the immature stage malic acid is predominant and its concentration is progressively reduced increasing that of citric acid [42,43]. 

In the Ibericus landraces, some specific trends in the acidic profile could be identified. Tendral tended to accumulate higher levels of malic acid and lower levels of citric, Amarillo tended towards lower malic acid contents. This trend might not be generalizable, as Albuquerque et al. [44] did not identify significant differences in the citric and malic acid contents in Portuguese accessions of Tendral and Pele de Sapo. In any case, it seems evident that within each landrace it is possible to identify accessions with differing levels of acid accumulation, which would be useful for the development of breeding programs of sweet melon cultivars with an acidic profile, leading to a very unique sensorial profile [41,45].

Differences were also found in the sugar profile of the landraces. As expected, due to the aforementioned lower SSC values of Tendral melons, sucrose and hexoses contents were lower, a configuration that might be related to the ripening process, as it has been shown that hexoses content in Tendral melons increases during long-term conservation due to starch and sucrose hydrolysis [38]. Interestingly, higher sucrose and hexoses accumulation was found in Rochet melons leading to higher sucrose equivalents values. This variable that weighs the sweetening power of each sugar has, in other crops, a higher relationship with sweetness perception [46], a key factor defining melon taste [45]. In this context, further analysis of this profile would enable the establishment of breeding program targets in the future. 

Regarding the VOCs profile, the Flexuosus and Chate melons exhibit a completely different profile compared to the Spanish Ibericus landraces, with high contents of aldehydes and alcohols and low contents of esters. This profile is highly related to the moment of evaluation, as fruits were harvested following commercial practices in the area, at immature state, and in melons, VOCs accumulate during the ripening process [47]. Other works have evaluated the Flexuosus VOC profile at the mature stage and, considering the climacteric nature of these fruits, they were characterized by a moderate to high content in ethyl esters [48]. Nonetheless, Tang et al. [49] and Chen et al. [50] both analysed the VOCs of the “Cai Gua” Flexuosus melon, harvested the fruits at commercial maturity, and found a similar VOCs profile, rich in aldehydes. Flores-León et al. [7] analysed the aroma of a Spanish Flexuosus melon at a commercial maturity, finding that the main VOCs were aldehydes, followed by alcohols, and that the aldehydes were (E,Z)-2-6-nonadienal, followed by E-2-nonenal, hexanal, and benzaldehyde. In our study, among the main aldehydes found at the immature stage (E,Z)-2,6- nonadienal, (E)-2-nonenal, nonanal, and (Z)-6-nonenal stood out. Among them (E,Z)-2,6- nonadienal and (E)-2-nonenal have an important impact on melon aroma [51]. (E,Z)-2,6- nonadienal is reported to contribute to a cucumber-like/green odour, while (E)-2-nonenal provides a fresh/green odour. Only one Chate accession was evaluated, and some level of variability might be expected, as in the case of Flexuosus. Nonetheless, our results seem to point out that Chate melons would tend to show a richer VOC profile compared to Flexuosus melons, especially in the accumulation of aldehydes and alcohols. 

The differences in the VOC profile between the Flexuosus, Chate, and Ibericus groups were also evident in the different network correlation analyses obtained with each group. Esteras et al. [48] obtained a general network using a wide spectrum of melon variability where acetate and ethyl esters were highly correlated, a result also obtained by Freilich et al. [52] with a RILs collection derived from a cross between the Momordica and Cantaloupensis group or by Perpiña et al. [53] in an ILs collection introgressing the Makuwa genome, using Cantalopuensis as the recurrent parent. This intercorrelation was also found in Ibericus melons, but in the Flexuosus and Chate melons acetate esters and butyrate esters, despite showing high correlation values within groups that are less intercorrelated. In this group, alcohols and aldehydes show high within-group and between-groups correlations, but the between-group correlation in the Ibericus melons is lost. These differences are probably related to the different harvesting dates for each material: immature in the case of Flexuosus and Chate and mature in the case of Ibericus melons.

The levels of esters detected in the Ibericus landraces were low, a characteristic typical of non-climacteric melons. Previous works have shown that Ibericus melons have levels of esters in the rind similar to Cantaloupensis climacteric melons, but the contents are considerably reduced in the flesh [54]. Indeed, when wide collections of melon germplasm have been analysed for their VOC profile, Inodorus melons, which include the Ibericus group, are grouped with sweet and non-sweet non-climacteric melons with low VOCs accumulation and non-sweet climacteric melons, such as Flexuosus, Chate, Ameri, and Momordica. In a study by Esteras et al. [48], Amarillo, Tendral, and Blanco landraces were grouped in a cluster characterized by fewer lipid-derived VOCs and higher acetate esters, while Piel de Sapo melons appeared in another subcluster characterized by higher amounts of linoleic acid derivatives, such as pentanal and hexanal. Our results, with a higher number of populations per landrace, confirm these trends. Although the spectra of variation in the VOC profile of different landraces tend to overlap due to the high variability found, it seems clear that Amarillo, Rochet, and Blanco tend to accumulate higher amounts of esters, while Piel de Sapo melons are richer in aldehydes.

Interestingly, within each landrace a high level of variation is present. For example, in Piel de Sapo accessions, 8PS, 10PS, 09PS, 12PS, and 07PS are rich in aldehydes, especially (E,Z)-2,6-nonadienal, as compared to 11PS and 03PS, which accumulate lower levels of these compounds and higher levels of alkyl and acetate esters. (E,Z)-2,6-nonadienal provides green and cucumber-like aromatic notes (http://www.thegoodscentscompany.com/) (accessed on 9 June 2022), while esters are typical of aromatic climacteric melons. Indeed, esters tend to contribute to fruity, sweet, and melon-like notes, whereas aldehydes and alcohols tend to contribute green, fresh, and cucumber-like notes [51]. 

In other crops, such as tomato, an autogamous species, it has been demonstrated that spontaneous cross-pollination and seed-mixing during centuries of cultivation would generate variability, which is then reduced by farmer selection in external morphological traits, defining each landrace, but the variability would be maintained in internal attributes, such as those related to fruit quality [55]. Indeed, in tomatoes it has been proven that the spectra of variation of landraces tend to overlap, identifying general trends in each landrace but maintaining a high level of variability [56]. As in our case, accessions of different landraces appear clustered together in genomic analysis, suggesting a common genetic background. In the case of the tomato, it is known that most morphological traits defining the external appearance of the landrace are controlled by a highly limited number of genes [56]. Consequently, despite detecting clear morphological differences, genetic differences are not evident in genomic analysis. Spontaneous crosses and consequent strong farmer selection pressures applied to redirect segregating populations have been described in an autogamous species, such as the tomato. Therefore, it would be reasonable to expect a similar evolution in a cross-pollinating species, such as the melon. Indeed, our data suggest that the different Ibericus landraces share a common genomic background with subtle differences, and that, although general trends can be identified, a high degree of variation is observed from a metabolomic point of view. This profile is consistent with the application of strong selection pressures to recover the external traits defining landrace morphology, following spontaneous crossings between different landraces grown in the same area. As in other crops, such as tomato, it would be necessary to develop depuration programs in order to tackle on-farm in situ conservation strategies focused on high quality markets. Indeed, it would be crucial to select and promote those accessions with a sugar, acid, and volatile profile that maximizes the the potential quality of the landrace. 

## 4. Materials and Methods

### 4.1. Plant Material

A collection of 47 melon accessions representing Spanish diversity were included in this study (Supplementary Table S5). They belonged to landraces of the Ibericus (33), Flexuosus (5), and Chate groups, and were provided by Universitat Politècnica de València germplasm bank (http://www.upv.es/contenidos/BGCOMAV/indexc.html) (accessed on 9 June 2022). Several external controls were also included: “T111”, a Piel de Sapo breeding line (Semillas Fito S.A.); the introgression line “calc8-1” [57], derived from an ILs population of a Momordica accession into a PS cultivar; the Chate accession (Chate-Car [14], coded as “41CHA”) as an example of another traditional non-sweet melon cultivar; the accession 39AL, a Flexuosus melon from Algeria representing an alternative morphological variant; one accession from the Ameri group (Am-NesviGeor [23], coded as I156), three accessions of Momordica group (La-KankMali [23], coded as I176; two individuals of IC274006 [58] (coded as IC-3 and IC-4); one of the Kachri group (WM7 [59]); and one from the Agrestis group (Trigonus [60], coded as Tri).

### 4.2. Experimental Design

Melon plants were cultivated in the greenhouse facilities located in the “Fundación Cajamar” in Paiporta, Valencia (Figure 1) (39°25′05.8″ N 0°25′03.4″ W). A total of 3 plants per accession were transplanted at the start of April. The plants were transplanted onto substrate bags of 29 kg with a 3:7 coconut chips to coconut fibre ratio. Irrigation was performed using drip systems. Nutrients were supplied to the plants through the irrigation water supply. The plants were pruned so as to regulate the vegetative growth and flowering of the plants. Melon fruits were collected when fruits achieved commercial maturity. A total of 5 fruits per accession were collected and characterized.

### 4.3. DNA Extraction and Genotyping-by-Sequencing Libraries

Prior to transplantation, leaf tissue from each melon accession was collected and frozen with liquid Nitrogen and stored at -80°C. The DNA extraction was performed following the CTAB protocol [61]. The extracted DNA was resuspended in MilliQ water. GBS libraries were then prepared, employing the restriction enzyme MsII, using the Illumina NovaSeq 6000 SP FC platform (Illumina Inc., San Diego, CA, USA) in the LGC Genomics GmbH (Berlin, Germany), following the procedure by Elshire et al. [62]. They raw reads were then quality-filtered, adapted, enzyme-clipped, and processed (2 × 150 bp).

### 4.4. SNP Calling and Analysis

The high quality paired-end reads were first mapped to the latest version of Melon reference genome (v4.0), available at melonomics.net [63], employing Bowtie2 v2.3.4.1 [64] with the “--very-sensitive” option. This ensures a slower but more sensitive and accurate mapping. The resulting mapping files (SAM format) were then converted into BAM format with Samtools v1.11 [65]. Freebayes v1.3.4 [66] was used to call the SNPs, setting a minimum mapping quality cut-off of 40, minimum base quality of 20, minimum base count of 10, and eliminating indels. This raw SNP file was further filtered using Vcftools [67], with a minor allele frequency (--maf 0.01), and maximum missing count (--max-missing-count 4). The variant calling file was further analysed using the SnpEff program [68] to view the SNPs with the highest effect.

### 4.5. Population Structure

Firstly, a principal component analysis (PCA) was performed employing Tassel 5 software for Windows [69] and the results visualized using CurlyWhirly Software for Windows. To investigate the population structure of the 47 accessions, an ADMIXTURE [70] analysis was performed. The admixture-linux-1.3.0 was run by employing the default parameters with an unsupervised mode with K = 1 to 10. The cross-validation error for each K was obtained with the -cv option, identifying the best suitable modelling. The cross-validation error K graph and Ancestry Q files were plotted using R.

### 4.6. Phylogenetic Relationship

All SNPs were concatenated into a single pseudo-sequence for all the 46 accessions. ClustalW 2.1 [71] for Linux was employed to perform an alignment of the sequences. PAUP* [72] version 4 for Windows was used to select the best substitution model, resulting in the choosing of model GTR+G nucleotide substitution model. Finally, the aligned sequences were introduced in RAxML v8.2.12 [73] to create the maximum-likelihood tree, inputting the substitution model selected (-m GTRGAMMA), indicating the outgroup, in this case being Trigonus accession (-o “Tri”), and a bootstrap of 1000 (-N 1000). Finally, the program MEGAX [74] for Windows was employed to visualize and perform a cut-off low scoring bootstrap branches (cut-off at 50% bootstrap). Evolview [75] was used to edit the tree.

### 4.7. Linkage Disequilibrium Decay

To understand the variability between the different melons, a pairwise estimate of the linkage disequilibrium (LD) was performed employing the program PopLDdecay [76]. The LD was analysed for the total melons studied, as well as 3 different subgroups, the Ibericus melons, Flexuosus, and Chate melons, and the exotic germplasm. LD values (r^2^) with respect to the genetic distance (kbp) were plotted. A maximum distance between loci of 1500 kbp was used to study the LD decay.

### 4.8. Melon Characterization

The collection of Spanish accession was characterized. A total of 5 melons per accession were harvested at their commercial maturity state. Fruits were characterized for fruit weight (FW in g, measured with a digital scale), fruit length, diameter, and cavity (FL, FD, and FC, in cm, measured with a ruler), rind thickness (in mm, with a Vernier calliper), rind and flesh firmness (RF and FF, measured with a penetrometer in kg/cm^2^), fruit pH (universal pH indicator paper), and soluble solids content (SSC, °Brix, measured with some drops of juice using a hand-held Pocket refractometer (PAL-α), Atago CO., LTD, Tokyo, Japan). Finally, both the fruit flesh and rind colours were measured in Hunter L, a, and b coordinates (CR-400 colorimeter, Konica Minolta, Inc., Tokyo, Japan). Additionally, out of the 5 fruits, 3 were sampled for their sugar and acid content.

### 4.9. Metabolomic Analysis

Accessions of the Spanish landraces were also analysed by determining sugar and acid and volatile profiles. For that purpose, three fruits were randomly selected. In the Ibericus group, a 5 cm cross-section of the fruit in the equatorial area was obtained, rind was discarded, and the edible flesh was homogenized (Silent Crusher M; Heidolph, Schwabach, Germany) and frozen at −80°C until analysis. In the case of the Flexuosus and Chate groups, the whole fruit was sampled after discarding the rind. Aliquots were used to measure sugars (glucose, fructose, and sucrose) and organic acids (malic, citric, and glutamic acids) employing capillary electrophoresis and volatile organic compounds (VOCs). 

For sugar and acid analysis, the methodology used in Martí et al. [77] was followed using an Agilent 7100 system (Agilent Technologies, Waldbronn, Germany). Samples were defrosted in the dark and centrifuged at 510 revolutions g^−1^ for 5 min. The upper phase was diluted with deionized water at a 1:20 ratio. It was then filtered employing 0.22 µm membranes (Costar^®^ Spin-X^®^, Corning, Amsterdam, The Netherlands). Fused-silica capillaries (Polymicro Technologies, Phoenix, AZ, USA) with dimensions of 50 µm internal diameter, 363 µm external diameter, 67 cm total length, and 60 cm effective length were used for the separation process. Before using for the first time, the capillaries were conditioned by flushing 1 mol L^−1^ NaOH at 95 kPa for 5 min at 50 °C, 0.1 mol L^−1^ NaOH for 5 min at 20 °C, and then 10 min with deionized water. At the beginning of each sequence, the capillary was flushed for 30 min at 20 °C with the running buffer, which consisted of a 20 mmol L^−1^ 2,6-pyridine dicarboxylic acid and 0.1% w:v hexadimethrine bromide solution adjusted at pH 12.1. Between samples, the capillary was flushed with 58 mmol L^−1^ sodium dodecyl sulphate (2 min) and running buffer (5 min). Between runs, the capillary was flushed with 58 mmol L^−1^ SDS (2 min) and BGE (5 min). Samples were injected hydrodynamically at 3400 Pa for 10 s and separations were performed at −25 kV and 20 °C. Absorbance was measured at 214 nm. Results were expressed in g kg^−1^ of fresh weight. Sucrose equivalents were calculated as the sum of sugar contents weighed with their sweetening power [78].

### 4.10. Volatile Organic Compound Analysis

The analysis of VOCs (Supplementary Table S6) was adapted from that described in Perpiñá et al. [53]. Solid Phase Extraction (SPE) cartridges used for retention were conditioned with 5 mL of diethyl ether and 5 mL of n-hexane and then dried for 10 min. Frozen samples were defrosted in the fridge. Once thawed, 30 g of the sample were weighed into a 150 mL Erlenmeyer flask with a stopper. The extraction process was carried out employing a Purge and Trap headspace system, with the SPE cartridge for the outlet tube and N2 gas for the inlet tube. The samples were extracted for 49 min at 40 °C using magnetic agitation and nitrogen flow of 1.6 mL min^−1^. Afterwards, 5 mL of each diethyl ether–hexane 1:1 (v:v) solution and diethyl ether were used to elute the cartridges. Finally, the collected elution solvents were evaporated to 0.5 mL at 35 °C under a nitrogen flow. Resulting extracts were divided into two aliquots in sealed gas chromatography (GC) vials and frosted at −20 °C until analysis. VOCs chromatographic analysis was performed employing a TQ-GC gas chromatography system from Waters (Milford, MA, USA), equipped with a Supelcowax column of 30 m × 0.25 mm × 0.25 µm (Sigma-Aldrich, San Luis, MO, USA). Helium was used as carrier gas at a flow of 1 mL/min. The injection was performed in splitless mode, injecting 1 μL of sample at 280 °C. The temperature program started at 40 °C (5 min), was then raised to 160 °C (4 °C min^−1^), and continued to 250 °C (30 °C min^−1^), which was maintained for 2 min. The mass spectra were acquired in selected ion monitoring (SIM) mode using the characteristic ions for each compound. Electron ionization in positive mode was used at a temperature of 250 °C and 230 °C for the interphase and the ion source, respectively.

### 4.11. Statistical Analysis

StatGraphics Centurion version 17.2.04 for Windows and IBM SPSS Statistics 25 for Windows were used to perform the analysis. R v4.1.2 for Windows, with usage of packages “ggplot2” [79]. Correlation networks analysis was conducted with the Expression Correlation plug-in (www.baderlab.org/Software/ExpressionCorrelation) (accessed on 9 June 2022) for the Cytoscape software v3.9.1. [80]. Nodes represent each individual volatile compound. Positive correlations were indicated in red edges and negative in blue. Principal component analysis (PCA) of morphological data and VOCs accumulation were performed using S-Plus v. 8.01 for Windows (Insightful Corp., Seattle, WA, USA). A biplot representation was then obtained, including the scores of data points and the loadings of each variable for each principal component.

## Data Availability

Datasets available at DOI:10.5281/zenodo.6728047. Further information available at request from the corresponding author.

## Supplementary Materials

The following supporting information can be downloaded at: https://www.mdpi.com/article/10.3390/ijms23137162/s1.
